# Editorial

**Published:** 1901-12-15

**Authors:** 


### EDITORIAL.

With this number the fifty-fifth volume of The Dental Register is made up. That it may creditably take its place alongside of its predecessors is our most earnest wish. That we have accomplished just what we planned, during the past year, we can not say. Circumstances have much to do with the life and development of individuals, and much more accidental circumstances will continuously change the best made plans of conducting a public magazine, which depends largely on its readers for its contents. We are often tempted to cut loose from all usual methods of securing copy, and lay out a special line of work and follow it closely; and let others print the current literature of our constituents. But after mature reflection we have concluded that our monthly repast will be more digestible if our readers are freely admitted to contribute any delicacy or substantial which they may have to enjoy and which they wish to contribute for the general good. We have tried during the year, and succeeded very well, we think, in presenting the transactions of the dental societies in which our subscribers are most interested; at the same time we have taken notice of the more important contributions to dental periodical literature. We shall continue the same policy, so far as we now know, during the coming year, and, while not promising so much as some of our worthy and ambitious contemporaries, we hope to improve the quality and value of the next volume, because we trust we have learned some things that will be helpful. We solicit the influence and co-operation of our readers not for our sakes, but for the good of all concerned. We promise to do our part as faithfully as we are able. Will you not do as much?

---

"*Now is the time to subscribe for the new year*" meets us as a prominent announcement on every magazine which comes to our table. Why should we subscribe? Because we should read and keep posted as to what is passing along with our times. Someone has said that the dental profession is not a reading one. Whether this is so or not we can not say, but we do know that there is no good reason why it should not read. It has many good magazines, some better, others not so good, perhaps, but all worth reading; and they are easily secured. A great many, we suspect, are actually given away by rich and ambitious publishers. Fifteen dollars will pay for eleven of the best journals published in this country; and five dollars, judiciously expended, will pay for enough journals to keep any one well in touch with passing events. Several good journals can be obtained for one dollar per year each. So there is really no valid excuse for any dentist not reading. At an early date, then, look the field over and order your next year's reading.

---

A new dental journal, called *The Dental Era*, is to begin publication at St. Louis next month. It will be in charge of Drs. J. H. Kennerly and Herman Prinz. If any one can make a venture of this sort succeed at St. Louis, we know of none more likely than the above gentlemen. We welcome the new journal and commend it to our readers.

